# Chronic Liver Disease, Not Everything Is What It Seems: Autoimmune Hepatitis/Primary Biliary Cholangitis Overlap Syndrome

**DOI:** 10.7759/cureus.51630

**Published:** 2024-01-04

**Authors:** Bruna Rodrigues Barbosa, Laurinda Pereira, Fátima Campante, Ana Paula Pona

**Affiliations:** 1 Department of Internal Medicine, Centro Hospitalar Barreiro Montijo, Barreiro, PRT

**Keywords:** overlap syndrome, autoimmune hepatitis/primary biliary cholangitis, liver cirrhosis, fibrosis, ascites, primary biliary cholangitis, autoimmune hepatitis

## Abstract

Overlap syndrome (OS) is a rare condition that shares characteristics of at least two other recognized diseases, whose early diagnosis impacts treatment decisions and prognosis since the unfavorable course of the OS seems to be worse than that of the diseases alone. OS in autoimmune liver diseases combines characteristic features of autoimmune hepatitis (AIH), primary biliary cholangitis (PBC), and primary sclerosing cholangitis. AIH is a chronic, inflammatory disease of the liver that occurs predominantly in females. The disease may start as acute hepatitis and progress to chronic liver disease and cirrhosis. PBC is characterized by a T-lymphocyte-mediated attack on small intralobular bile ducts. A continuous assault on the bile duct epithelial cells leads to their gradual destruction and eventual disappearance. The sustained loss of intralobular bile ducts causes the signs and symptoms of cholestasis and eventually may result in cirrhosis and liver failure. With treatment with ursodeoxycholic acid, the majority of patients now have normal life expectancies. The authors report a subtype of OS, i.e., AIH-PBC overlap, characterized by elevated serum transaminases, cholestasis markers, antimitochondrial antibodies (AMAs), and histological findings compatible with AIH, including moderate-to-severe interface hepatitis. The authors present a clinical case referred for internal medicine consultation regarding a 73-year-old woman presenting pancytopenia and increased transaminases, along with weight loss, decreased appetite, and tiredness. Laboratory tests were positive for the following parameters: antinuclear antibody, anti-double-stranded DNA antibody, AMA, anti-glycoprotein-210, and anti-smooth muscle antibody (anti-actin). Computed tomography of the abdomen displayed chronic liver disease and evidence of small perihepatic ascites. The diagnosis was established with a liver biopsy revealing architectural alteration with severe advanced fibrosis, with bridges and parenchymal nodularity, and histological parenchymal changes of progressive chronic liver disease (chronic biliary disease/PBC) in the stage of cirrhosis. With proper treatment, the condition of the patient significantly improved.

## Introduction

Autoimmune hepatitis (AIH) is a chronic inflammatory liver disease of unknown etiology. It is thought that genetic predisposition, environmental factors, and failure of the native immune system may be involved in its development, leading to chronic inflammation of hepatocytes and subsequent liver fibrosis. It frequently affects women of all ages and is characterized by an increase in transaminases, hyperglobulinemia, circulating auto-antibodies, and portal inflammatory infiltration with interface hepatitis in the liver biopsy. Treatment is based on a combination of corticoids and azathioprine, which induces remission in the majority of patients, but 20% to 40% require second or third-line therapies (such as mycophenolate mofetil, cyclosporine A, or tacrolimus) due to intolerance or insufficient response (maintenance of symptoms and a weak reduction of laboratory parameters). This study reviews the most vital aspects regarding the diagnosis and treatment of AIH, emphasizing the challenges faced in clinical practice [1]. Patients with certain cholestatic liver diseases, such as primary biliary cholangitis (PBC), have clinical and serological features suggesting the presence of AIH and respond to immunosuppressive therapy [2]. In addition, PBC-AIH overlap syndrome (OS) is a clinical entity characterized by the simultaneous occurrence of both conditions in a patient, which is reviewed in this article. Immunological and clinical characteristics suggest a variant form of AIH in 1% to 14% of patients with PBC [3-5].

## Case presentation

A 73-year-old autonomous woman had a past medical history of arterial hypertension, hip osteoarthritis (bilateral total hip prosthesis in 2001), and gonarthrosis. The patient was chronically medicated with irbesartan and hydrochlorothiazide (300 mg and 12.5 mg, respectively), nimesulide (100 mg), and tramadol with paracetamol (37.5 mg and 325 mg, respectively), as needed. The family doctor referred the patient to the medicine consultation after presenting for more than 10 years with a discreet chronic anemia (hemoglobin between 11 and 12 g/dL) without apparent establishment of the cause, a slight increase in transaminases (around 40 to 70 U/L), and fluctuating gamma-glutamyl transpeptidase (GGT) (50 to 100 U/L) at least since 2011. The first evidence of a more significant increase in transaminases occurred in 2021 (Table 1). An abdominal ultrasound was requested for clarification, revealing only a slight steatosis infiltration. In January 2022, a more pronounced decrease in hemoglobin occurred along with leukopenia, thrombocytopenia, and a new increase in transaminases (Table 1). The patient took oral iron supplementation (previous iron levels: 39 µg/dL) and returned in May, reporting weight loss (about 6 to 7 kg in three months) associated with loss of appetite and tiredness. Upon reassessment, the patient exhibited an improvement in hemoglobin (11.3 g/dL); however, there was a further worsening of the remaining parameters, with an increase in total bilirubin.

**Table 1:** Laboratory tests AMA: antimitochondrial antibodies; ANA: antinuclear antibodies; anti-gp210: anti-glycoprotein-210 antibodies; ASMA: anti-smooth muscle antibodies (anti-actin); HIV: human immunodeficiency virus.

Laboratory tests	2021	Jan/2022	May/2022	Nov/2022	Ref. values
Hemoglobin (g/dL)	-	10.7	11.3	10.7	13–17
Leukocytes/μL	-	3,800	-	3,500	4,000–11,000
Platelets/μL	-	140,000	136,000	107,000	150,000–400,000
International normalized ratio	-	-	-	1.12	0.8–1.2
Creatinine (mg/dL)	-	-	-	0.97	0.7–1.3
Urea (mg/dL)	-	-	-	50	<50
Albumin (g/dL)	-	-	-	3.0	3.4–5.4
Aspartate aminotransferase (U/L)	141	268	546	425	<34
Alanine aminotransferase (U/L)	134	225	499	274	<35
Gamma-glutamyl transpeptidase (U/L)	80	109	154	123	12–64
Alkaline phosphatase (U/L)	140	-	164	119	13–43
Total bilirubin (mg/dL)	-	-	1.44	2.3	0.1–1.2
Direct bilirubin (mg/dL)	-	-	-	1.2	<0.3
Lactate dehydrogenase (U/L)	-	-	-	343	140–280
C-reactive protein (mg/dL)	-	-	-	14	<0.5
Total cholesterol (mg/dL)	-	-	-	231	<200
Triglycerides (mg/dL)	-	-	-	79	<150
Protein electrophoresis	No monoclonal pattern				
Anti-beta2 glycoprotein	Normal				
Alpha-fetoprotein	Normal				
HIV, hepatitis B and C	Negative				
ANA, anti-dsDNA antibody, AMA, anti-gp210, ASMA (anti-actin)	Positive				
Anti-LKM-1 and anti-LC1 antibodies	Negative				

In consideration of these laboratory results, a new abdominal ultrasound was requested, which displayed a frankly dysmorphic liver in probable relation to evolved chronic liver disease with evident relative enlargement of the left and caudate lobes. The ultrasound also demonstrated rhombus and wavy contours and diffuse granularity of the parenchyma coexisting with the distortion of the intrahepatic vascular network in relation to structural changes. It was also observed that the hilum diameter of the portal vein trunk was at the upper limit of normality, splenic vein ectasia, and splenomegaly. In view of these alterations, a new analytical study was requested with negative alpha-fetoprotein (7.10 ng/mL), negative serologies for human immunodeficiency viruses (HIV), venereal disease research laboratory, hepatitis B and C, positive immunoglobulin G (IgG) antibodies for hepatitis A, coagulation without changes, and a sedimentation rate of 87 mm/h.

**Figure 1:**
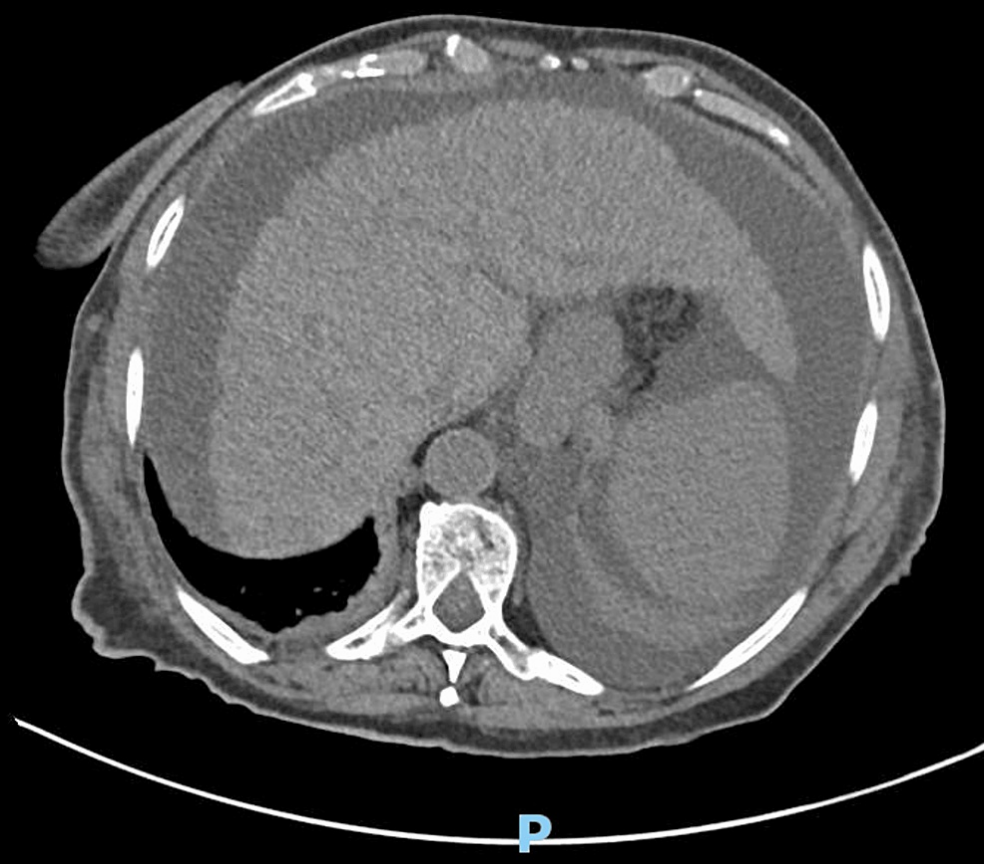
Abdominal computed tomography (axial section) Liver characteristics of chronic liver disease and evidence of small perihepatic ascites.

For etiological clarification, a computed tomography scan of the abdomen was also performed, revealing bosselated contours and preserved dimensions in the liver, slight calcifications in the capsule of the spleen, and very incipient dilatation of the central intrahepatic bile ducts without evidence of bile duct dilatation (Figure 1).

In this context, an internal medicine consultation was requested for the etiological study of this chronic liver disease. The patient had no complaints and denied ethanol consumption or pruritus. A complete evaluation for autoimmunity and magnetic resonance imaging was performed. Initially, because PBC was suspected due to compatible imaging changes, the patient started ursodeoxycholic acid (UDCA). Laboratory tests were positive for the following parameters: antinuclear antibodies (ANA: 3.7 U), anti-double-stranded DNA (anti-dsDNA) antibody (626.9 IU/mL), antimitochondrial antibodies (AMA), anti-glycoprotein-210, and anti-smooth muscle antibody (ASMA) anti-actin (Table 1). The abdominal magnetic resonance imaging revealed a prominent left lobe of the liver with irregular and crenated contours in relation to known chronic liver disease, with signal heterogeneity. In addition, imaging found a distended gallbladder without noticeable parietal or endoluminal changes. Further, the scan found splenomegaly (about 15 cm), a significant sliding gastric hernia, perihepatic and perisplenic ascites, and several prominent ganglia in the hepatic hilum (the largest measuring about 9 mm on the short axis) without enlarged lymph nodes.

**Figure 2:**
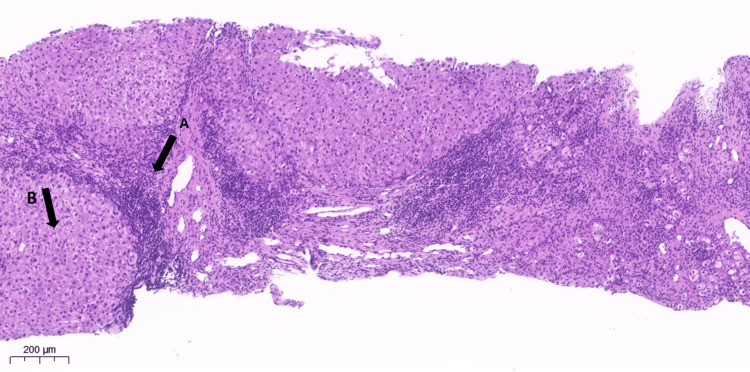
Liver biopsy Anatomopathological diagnosis: (A) architectural alteration with severe fibrosis, (B) advanced with bridges and parenchymal nodularity, a definitive indication of cirrhosis, finding histological and parenchymal changes with progressive chronic liver disease (chronic biliary disease/primary biliary cholangitis, in the stage of cirrhosis) without malignant neoplasm.

The diagnosis of PBC-AIH OS was confirmed. A liver biopsy was observed with evidence of architecture, nodularity, and enlargement of the portal spaces due to intense fibrosis, with the formation of porto-central septa and an inflammatory infiltration consisting of mononuclear elements, bile-duct involvement, and lymphoid aggregate formation with activity at the interface (Figure 2). The upper digestive endoscopy revealed large, bluish varicose cords without red spots in the distal third of the esophagus, which collapsed upon insufflation, and the presence of a sliding hernia of about 3 cm. The findings are compatible with portal hypertension, which, when present, worsens the prognosis.

Currently, the patient's treatment includes UDCA, beta-blockers, diuretics, and corticosteroid therapy. The patient has improved clinically and laboratorially, with no evidence of edema. In addition, the patient has recovered some weight and mobility, with an improved quality of life.

## Discussion

Although AIH can present at any age and in all ethnic groups, it predominantly occurs in women [6-8]. For type 1 AIH, the female-to-male ratio is 4:1. The incidence is 0.9-2 per population of 100,000 per year, with a prevalence of 11-25 per 100,000 [7,9-11]. Moreover, OS occurs in 3% to 7% of patients with autoimmune liver disease [12]. Type 1 AIH is characterized by positive ANAs or ASMAs, and type 2 AIH is characterized by positive anti-liver kidney microsomal type 1 or anti-liver cytosol type 1 antibodies [13].

Patients who have histologic features of AIH but have the serologic findings of PBC, sometimes referred to as AMA-positive AIH, appear to be almost identical to those observed in type 1 AIH [14]. In addition, AMA is the serological hallmark of PBC. Patients with AIH-PBC OS were more likely to have concomitant AMA/anti-dsDNA seropositivity than patients with pure AMA-positive PBC. Moreover, AMA/ANA is positive in 60% of these overlap patients, and such patients respond to combination therapy with UDCA and glucocorticoids [15]. Moreover, AIH superimposed on PBC can result in rapid progression to cirrhosis and liver failure [4]. In contrast, patients with OS were more likely to develop esophageal varices, gastrointestinal bleeding, ascites, and liver failure compared with patients with more classical PBC [16]. Further studies are needed to determine whether differences exist in the natural history of the disease or the therapeutic response.

Glucocorticoids and UDCA have been recommended for the treatment of patients with AIH-PBC OS whose liver histology is consistent with PBC [17]. Combining UDCA and corticosteroids has been suggested to have a synergistic effect [18]. UDCA, by accelerating bile acid enterohepatic circulation, has cytoprotective, anti-apoptotic, membrane-stabilizing, and anti-inflammatory effects. Glucocorticoids control the inflammation in the liver, thereby preventing further scarring. Combination therapy with UDCA and immunosuppression appears more effective than UDCA alone in improving or stabilizing liver histology, with up to seven years of follow-up in patients with strictly defined AIH-PBC syndrome [19]. An alternative approach is to start with just UDCA and add corticosteroids if UDCA therapy has not induced an adequate biochemical response within an appropriate period (three months to one year, according to different references). Steroid-sparing agents should be considered for patients who require long-term immunosuppression.

A reasonable approach to the treatment of patients with AIH-PBC OS is a trial of corticosteroids. Although only a minority of patients respond, biochemical and histological improvement may be achieved. If values of serum aminotransferases and alkaline phosphatase do not improve, corticosteroids should be discontinued and treatment initiated with UDCA (13 mg/kg/day). In addition, UDCA and azathioprine may be added as steroid-sparing agents in patients who respond to corticosteroids. A follow-up liver biopsy is often conducted after one to two years of treatment to document histological improvement in responding patients [2].

Although difficult to distinguish clinically in its early stages, AIH-PBC OS should be considered when a patient presents with the diagnostic criteria of both diseases [20]. The diagnostic criteria for AIH-PBC OS according to the Paris criteria shall contain at least two of the three selected criteria for PBC and AIH, respectively: alkaline phosphatase > 2x of the upper normal value (UNV) or GGT > 5x UNV, AMA positive, chronic granulomatous cholangitis at liver biopsy; and alanine aminotransferase > 5x UNV, ASMA or IgG > 2x UNV, and interface hepatitis (moderate/severe) at liver biopsy. The Paris criteria are 92% sensitive and 97% specific for PBC-AIH OS [20]. In this case, the patient was positive for ANA, ASMA (anti-actin), and AMA, with histological findings corroborating OS.

## Conclusions

This clinical case illustrates the complexity of this disease and its diagnostic process in an attempt to identify its etiology and start treatment as soon as possible. This pathology involves multidisciplinarity, with the internist being the patient manager and cooperating with other specialties, such as immunology, hepatology, and gastroenterology. Early diagnosis and intervention for this condition are required for a better outcome. Maintaining close monitoring, probably with follow-up until confirmed histopathological remission, is vital. In clinical practice, investigating beyond the obvious and considering the possibility of these diagnoses is critical.
